# Lipid metabolism dysregulation in late-life depression: peripheral metabolic disturbance, central lipid alterations, and dietary modulation

**DOI:** 10.3389/fnut.2026.1926500

**Published:** 2026-09-09

**Authors:** Yao Gao, Han-Ni Li, Ze-Kun Li, Jian-Zhen Hu, Xin-Zhe Du, Xin Yan, Sha Liu

**Affiliations:** 1 Department of Psychiatry, First Clinical Medical College, First Hospital of Shanxi Medical University, Taiyuan, China; 2 Shanxi Key Laboratory of Artificial Intelligence Assisted Diagnosis and Treatment for Mental Disorders, First Hospital of Shanxi Medical University, Taiyuan, China; 3 Nursing College, Shanxi Medical University, Taiyuan, China; 4 Drug Clinical Trial Institution, First Hospital of Shanxi Medical University, Taiyuan, China

**Keywords:** depression, dietary lipids, late-life depression, lipid metabolism, lipidomics

## Abstract

Late-life depression involves neurodegeneration, inflammation, metabolic disruption, and psychosocial stress. Lipid metabolism has drawn increasing attention in this field. This review examines three interconnected aspects: peripheral metabolic disturbance, potential central lipid alterations, and dietary lipid regulation. Peripherally, adverse lipid profiles and lipidomic shifts correlate with depressive symptoms, cognitive decline, and cardiometabolic load. Mechanisms include inflammation, membrane remodeling, mitochondrial dysfunction, and oxidative stress. Centrally, the disorder may involve aberrant membrane phospholipid metabolism, brain region–specific lipid alterations, and apolipoprotein E–mediated transport disruption, affecting myelin integrity, synaptic plasticity, neuroinflammation, and cerebrovascular function. Evidence regarding dietary lipids remains mixed: omega-3 supplementation has not shown a consistent preventive benefit, whereas high-fat, fried, and Western dietary patterns have been associated with depressive outcomes in observational studies. However, much of the current evidence is extrapolated from MDD or general populations, limiting its direct applicability to late-life depression. Future work should combine longitudinal designs, multi-omics, neuroimaging, and intervention studies to establish causality and identify biomarkers and tailored strategies.

## 1 Introduction

Late-life depression (LLD) generally refers to depressive syndromes occurring in older adults, typically defined using DSM or ICD criteria (1). Although an age threshold of 65 years is commonly used, some studies include adults aged 60 years and above. LLD is clinically heterogeneous and may occur alongside chronic medical illness, cognitive impairment, and disability. It is estimated that 332 million individuals worldwide are affected by depressive disorders (2). In the general population, the GBD-estimated prevalence of depressive disorders is approximately 4.0%, with a prevalence of approximately 5.7% among adults (4.6% in men and 6.9% in women), rising to 5.9% among those aged 70 years and older (3). A separate meta-analysis reported an overall prevalence of major depressive disorder (MDD) of 13.3% among older adults (11.9% in women and 9.7% in men) (4). However, these estimates were derived using different methodologies, with the GBD value based on modeled estimates and the MDD prevalence derived from a meta-analysis of clinical diagnostic and screening studies. As global population aging accelerates, the prevalence of LLD is expected to increase further, imposing heavy burdens on healthcare systems and socioeconomic structures. Currently, the pathogenesis of LLD remains unclear and is considered to result from multiple factors including genetic susceptibility, vascular pathology, neurotrophic factor imbalance, neuroinflammation, neuroendocrine disorders, aging, and degenerative changes (5). In recent years, metabolic disturbances, particularly abnormal lipid metabolism, have received growing attention in relation to LLD progression, potentially offering new research directions for elucidating the pathogenesis of this disorder.

Lipids are not only fundamental to energy storage and cell membrane structure but also are critical to brain structure and function, including myelination, synaptic plasticity, and the regulation of neuroinflammation. Lipid metabolism, the body’s synthesis, breakdown, and utilization of lipids, spans digestion, absorption, transport, storage, and conversion into energy and other biomolecules (6). Studies have found significant lipid metabolism disturbances in the anterior cingulate cortex of patients with MDD, characterized by elevated levels of free fatty acids and lysophosphatidylcholine, as well as specific changes in glycerolipids (7). Additionally, genetic and metabolomic studies suggest that m6A methylation regulators METTL14 and RBM15 may exert antidepressant effects by modulating lipid metabolism (8). However, direct evidence specific to LLD remains limited.

**Figure 1:**
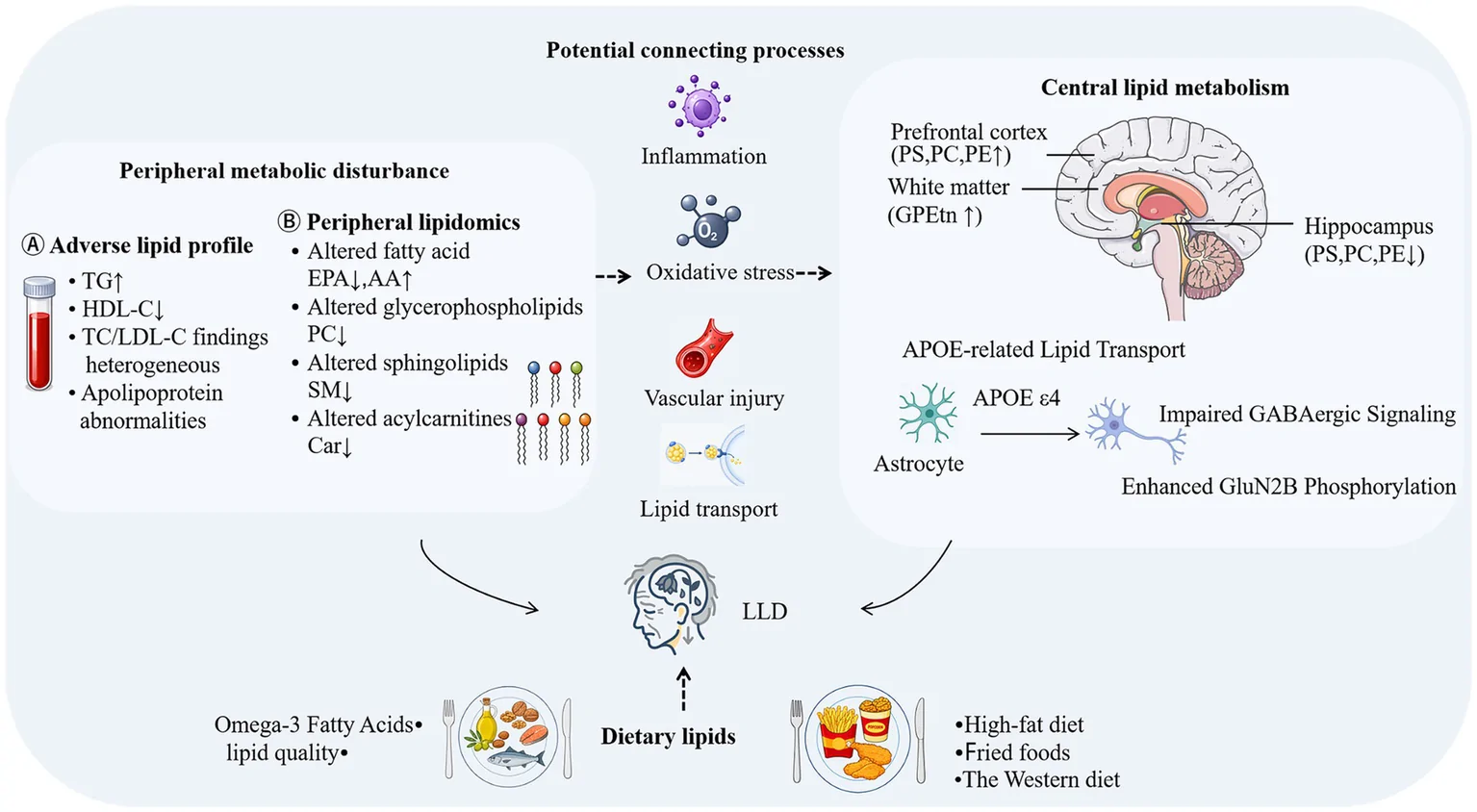
Proposed associations between lipid metabolism and LLD. Peripheral metabolic disturbance in late-life depression (LLD) is characterized by adverse lipid profiles, including increased TG and reduced HDL-C, heterogeneous findings for TC and LDL-C, apolipoprotein abnormalities, and lipidomic alterations involving fatty acids, glycerophospholipids, sphingolipids, and acylcarnitines. Inflammation, oxidative stress, vascular injury, and lipid transport may represent potential links between peripheral metabolic changes and central lipid-related processes. However, direct associations between peripheral and brain lipid alterations in LLD have not been established, and these links remain hypothetical. Limited evidence suggests that central lipid metabolism may involve region-specific phospholipid changes and APOE-related pathways. APOE ε4 may further affect neural function through impaired GABAergic signaling and enhanced GluN2B phosphorylation. Dietary lipid findings are mainly associative: evidence for omega-3 fatty acids is inconsistent, while high-fat, fried, and Western dietary patterns have been associated with adverse depression-related outcomes. HDL-C, high-density lipoprotein cholesterol; TC, total cholesterol; LDL-C, low-density lipoprotein cholesterol; EPA, eicosapentaenoic acid; AA, arachidonic acid; PC, phosphatidylcholine; SM, sphingomyelin; Car, acylcarnitine; PS, phosphatidylserine; PE, phosphatidylethanolamine; GPEtn, glycerophosphoethanolamine; APOE, apolipoprotein E; GluN2B, glutamate ionotropic receptor NMDA type subunit 2B.

Currently, LLD-related research lacks integration of peripheral and central lipid metabolism networks. Accordingly, this review provides an overview of the relationship between LLD and lipid metabolism, aiming to clarify the lipid-related biological basis of LLD and inform the development of novel diagnostic and therapeutic strategies (Figure 1).

## 2 LLD and metabolic abnormalities

LLD is a heterogeneous condition that includes early-onset depression (EOD) recurring in later life, late-onset depression (LOD), and depressive symptoms occurring as a prodromal feature or comorbidity of dementia. These forms of LLD may differ in their underlying pathophysiology. LOD is more often associated with vascular burden, executive dysfunction, and treatment resistance, whereas EOD is more closely related to a history of affective illness and personality factors (9, 10). Depression associated with cognitive decline or dementia may involve additional neurodegenerative processes. These differences suggest that vascular, neurodegenerative, genetic, and psychosocial factors may contribute to LLD to different degrees across subtypes. Such heterogeneity may also influence lipid metabolism and should be considered when interpreting lipid-related findings in older adults.

### 2.1 Age-related changes in lipid metabolism

Aging is accompanied by progressive changes in lipid metabolism, which provide an important background for interpreting lipid abnormalities in LLD. In peripheral tissues, aging is associated with increased adiposity, altered lipolytic responses, and reduced lipid clearance. These changes can affect circulating triglycerides, lipoproteins, and other lipid metabolites (11). Age-related changes are also observed in the brain. Human studies have shown that total brain lipid content gradually declines with age, together with changes in cholesterol, phospholipids, sphingolipids, and other myelin-related lipids (12, 13). These changes are not uniform across the brain. Reductions in cholesterol and phospholipids have been reported in the frontal and temporal cortices, hippocampus, and several other regions, while different lipid classes may show distinct age-related patterns (14). Such findings indicate that brain aging involves selective lipid remodeling rather than a simple overall increase or decrease in lipid levels.

These age-related changes are relevant to LLD because aging, vascular disease, metabolic disorders, and depression often coexist in older adults. Older age is also associated with a more persistent course of depression (15). Therefore, lipid abnormalities identified in LLD may partly overlap with changes related to aging and common age-related comorbidities.

### 2.2 Metabolic abnormalities in LLD

Many studies have focused on elucidating the contribution of metabolic dysregulation to the progression of LLD. Previous studies have shown that multiple components of metabolic syndrome, such as central obesity and hypertriglyceridemia, are associated with depression severity (16). However, the association between metabolic syndrome as an overall indicator and LLD prognosis remains inconsistent. The Whitehall II cohort study showed that metabolic syndrome itself was not associated with remission of depressive symptoms in old age, but low high-density lipoprotein cholesterol (HDL-C) and high triglyceride levels were associated with a reduced likelihood of symptom remission (17). A prospective study found that baseline metabolic syndrome predicted poor outcomes in LLD, and this association was mainly driven by abdominal obesity and HDL-C abnormalities (18). Other cohort studies failed to identify a significant link between metabolic syndrome and depressive outcomes (19, 20).

Overall, existing studies suggest that the association between metabolic syndrome as a whole and LLD remains inconsistent. But lipid-related indicators such as HDL-C and triglyceride levels have shown relatively more consistent associations with poor symptom recovery and prolonged disease course in some studies. Therefore, focusing on abnormal lipid metabolism may help to better understand the prognostic heterogeneity of LLD and its underlying biological mechanisms.

## 3 Peripheral lipid metabolism disturbances in LLD

### 3.1 Abnormal lipid profiles in LLD

Prospective studies provide the strongest direct evidence. In a Korean community cohort of adults aged ≥65 years, lower baseline HDL-C was associated with incident depression, and TC showed a U-shaped association with risk (21). A subsequent analysis of the same cohort suggested stronger HDL-C associations among 5-HTTLPR s-allele carriers (22). In the French ESPRIT cohort, low LDL-C was associated with prevalent depression and predicted incident depression in men, whereas low HDL-C was associated with prevalent but not incident depression in women (23). Together, these findings support an association between lipid homeostasis and LLD, but do not indicate a uniform direction across lipid fractions or sex.

Cross-sectional studies in older adults provide supportive but less causal evidence. In Chinese adults aged ≥60 years, high TC, TG, LDL-C, low HDL-C, and dyslipidemia were associated with depressive symptoms (24). CHARLS analyses further identified nonlinear inverse associations of the non-HDL-C-to-HDL-C ratio (NHHR) and remnant cholesterol (RC) with depressive symptoms, while longitudinal time-series analyses supported a more consistent association for NHHR than RC (25). These findings suggest that lipid-depression relationships in later life may be nonlinear and dependent on cardiometabolic context.

Evidence from younger or age-limited populations should be interpreted cautiously. Dyslipidemia was associated with greater symptom burden in first-episode drug-naïve MDD samples of adults aged 50–60 years and 18–45 years, but these studies were cross-sectional and not representative of conventional LLD (26, 27). In a large Swedish cohort, adverse metabolic markers, particularly high TG and low HDL-C, were linked with later psychiatric disorders, and several glucose and lipid abnormalities were detectable years before diagnosis (28). A Korean cross-sectional study of adults aged 40–64 years found an association between higher HDL-C and depressive symptoms, suggesting that results may differ by age group and study design (29).

Lipid-related abnormalities in LLD also include oxidative lipid damage. In the Health ABC study, higher urinary 8-iso-PGF2α was associated with depressed mood in older men but not women (30). In a clinical case–control study, patients with DSM-5-defined LLD had higher plasma free 8-isoprostane and lower glutathione peroxidase activity, and higher 8-isoprostane was associated with poorer cognitive performance (31). These findings support lipid peroxidation as a potential correlate of LLD, although both studies were cross-sectional and used different biological matrices and assays.

Directional differences in lipid–depression associations may be better understood as context-dependent rather than simply contradictory. In prospective cohorts of older adults, lower HDL-C or LDL-C was associated with prevalent or incident depression, whereas a middle-aged cross-sectional study found the opposite association for HDL-C. Moreover, the relationship does not appear to be linear: total cholesterol showed a U-shaped pattern, and composite indices such as NHHR and RC demonstrated nonlinear or threshold-dependent associations. Sex also appears to modify some relationships, with different lipid fractions showing distinct patterns in men and women. Although few studies have distinguished between EOD and LOD, available evidence suggests that onset subtype may further contribute to heterogeneity. Taken together, the direction and magnitude of lipid–depression associations likely depend on the specific lipid parameter, population age and sex, depression subtype, and study design, supporting a model of disturbed lipid homeostasis rather than a single adverse lipid profile (Table 1).

**Table 1:** Summary of studies on peripheral lipid metabolic abnormalities related to LLD.

Study	Design and population	Depression ascertainment	Lipid-related biomarkers	Key finding
Prospective cohort studies				
Kim et al., 2006 (21)	Prospective community cohort; 521 Korean adults aged ≥65 years without baseline depression	GMS B3 + AGECAT (confidence level ≥3); incident: present at follow-up but absent at baseline	HDL-C; TC; LDL-C; TG	Lower HDL-C was associated with incident depression; TC showed a U-shaped association.
Kim et al., 2011 (22)	Prospective analysis of the same Korean cohort as Ref. 21	GMS B3 + AGECAT (confidence level ≥3); 2-year follow-up; incident: present at follow-up but absent at baseline	HDL-C; TC; LDL/HDL ratio; 5-HTTLPR	Lower HDL-C was associated with prevalent and incident depression; especially among 5-HTTLPR s-allele carriers
Ancelin et al., 2010 (23)	7-year prospective ESPRIT cohort; 1,792 community-dwelling French adults ≥65 years	DEP = CES-D ≥ 16 or current major depression on MINI; incident DEP assessed during follow-up	LDL-C, HDL-C; 5-HTTLPR	Low LDL-C was associated with prevalent depression and predicted incident depression in men; low HDL-C was associated with prevalent, but not incident, depression in women
Cross-sectional studies				
Liang et al., 2014 (24)	Cross-sectional; 1,529 Chinese adults aged ≥60 years	GDS-15; elevated depressive symptoms defined as score ≥5	TC, TG, LDL-C, HDL-C; dyslipidemia	High TC, TG and LDL-C, low HDL-C, and dyslipidemia were associated with elevated depressive symptoms.
Gu et al., 2026 (25)	CHARLS-based cross-sectional and longitudinal analysis; Chinese adults aged ≥60 years	CESD-10; score ≥10 classified as LLD/depressive symptoms	NHHR, RC	NHHR and RC showed nonlinear inverse cross-sectional associations; longitudinal support was more consistent for NHHR.
Milaneschi et al., 2013 (30)	Cross-sectional analysis in a community cohort; 1,975 adults aged 70–79 years	Depressed mood = GDS-15 > 5 and/or antidepressant use	Urinary 8-iso-PGF2α	Higher urinary 8-iso-PGF2α was linked with depressed mood in older men.
Diniz et al., 2018 (31)	Cross-sectional case–control study; 77 LLD patients and 47 non-depressed controls	MINI + DSM-5 diagnosis of MDD; HDRS-21 used for symptom severity	Plasma free 8-isoprostane; GPx	LLD showed higher free 8-isoprostane and lower GPx activity; 8-isoprostane correlated with poorer cognition.

This table summarizes studies examining associations between peripheral lipid metabolic abnormalities and LLD. GMS B3, Geriatric Mental State diagnostic schedule (version B3); AGECAT, Automated Geriatric Examination for Computer Assisted Taxonomy; 5-HTTLPR, serotonin transporter linked promoter region; ESPRIT, Enquête de Santé Psychologique-Risques, Incidence et Traitement; DEP, clinical level of depression (CES-D ≥ 16 or current major depression on MINI); CES-D, Center for Epidemiologic Studies Depression Scale; MINI, Mini-International Neuropsychiatric Interview; GDS-15, 15-item Geriatric Depression Scale; CHARLS, China Health and Retirement Longitudinal Study; CESD-10, 10-item Center for Epidemiologic Studies Depression Scale; 8-iso-PGF2α, 8-iso-prostaglandin F2α; DSM-5, Diagnostic and Statistical Manual of Mental Disorders, 5th edition; HDRS-21, 21-item Hamilton Depression Rating Scale; GPx, glutathione peroxidase.

### 3.2 Lipidomic characteristics in LLD

#### 3.2.1 Peripheral lipidomic evidence in LLD

Compared with traditional blood lipid indicators, lipidomics can reveal the specific characteristics of peripheral lipid metabolism disturbances in patients with LLD at the molecular level with greater precision. Existing studies, which are predominantly observational, show that peripheral lipidomic abnormalities in LLD mainly involve fatty acids, glycerophospholipids, sphingolipids, and acylcarnitines (Table 2). These changes are not confined to single lipid molecules, but may collectively point to pathological processes such as inflammatory lipid imbalance, membrane lipid remodeling, impaired mitochondrial energy metabolism, and impaired neuroplasticity. Therefore, lipidomic research not only helps identify peripheral biomarkers for LLD, but also provides important clues for understanding the relationships among depressive symptoms, cognitive impairment, and aging-related neurological decline.

**Table 2:** Peripheral lipidomics-related evidence in LLD.

Study (author, year)	Design	Population	Sample and analytical methods	Main findings
Gao et al., 2025 (32)	Systematic review of 12 studies (10 cross-sectional; 2 cohort).	Studies of LLD and healthy controls	Serum, plasma, CSF, or urine; targeted/untargeted metabolomics using GC, GC–MS, or LC–MS/MS.	Total fatty acids, EPA, 9,12-octadecadienoate↓; AA↑; oleate findings were inconsistent.
Wang et al., 2026 (33)	Cross-sectional comparison of active LLD episodes versus remission	110 adults aged ≥55 years with clinically diagnosed LLD: 33 active episode and 77 remission	Fasting EDTA plasma; untargeted UHPLC-Orbitrap LC–MS metabolomics.	During episodes, Car(13:0), PC(P-16:0/22:6) and SM(d18:1/22:0) ↓. Car(13:0) and PC(P-16:0/22:6) were linked with depressive severity and cognitive performance.
Diniz et al., 2016 (35)	Exploratory cross-sectional case–control proteomics study	75 adults aged ≥65 years: 44 with remitted, DSM-IV-defined LLD and 31 never-depressed controls	EDTA plasma; Human DiscoveryMAP 250 + multiplex proteomic assay; 195 proteins analyzed.	ApoA-IV, FABP-liver, ApoA-II, and ApoA-I↓; ApoB, ApoE↑.

This table summarizes studies examining peripheral lipidomics-related alterations in LLD, including changes in fatty acids, acylcarnitines, glycerophospholipids, sphingolipids, and lipid transport-related proteins. CSF, cerebrospinal fluid; GC, gas chromatography; GC–MS, gas chromatography–mass spectrometry; LC–MS/MS, liquid chromatography–tandem mass spectrometry; EPA, eicosapentaenoic acid; AA, arachidonic acid; EDTA, ethylenediaminetetraacetic acid; UHPLC, ultra-high-performance liquid chromatography; LC–MS, liquid chromatography–tandem mass spectrometry; Car(13:0), tridecanoylcarnitine; PC, phosphatidylcholine; SM, sphingomyelin; Apo, apolipoprotein; FABP-liver, liver-type fatty acid-binding protein; ↑ increased; ↓ decreased.

Of these alterations, fatty acid dysregulation provides the clearest inflammatory signal. A systematic review of metabolomic findings in LLD, in which most included studies were cross-sectional, reported reduced total fatty acids, EPA, and 9,12-octadecadienoate, together with increased AA, whereas findings for oleate were inconsistent (32). This pattern suggests a shift from anti-inflammatory or resolution-associated lipid activity toward a more pro-inflammatory AA-related profile. Because EPA and AA are precursors of lipid mediators with opposing inflammatory tendencies, an EPA-AA imbalance may facilitate neuroinflammation, oxidative stress, and synaptic or neuronal dysfunction in LLD. Oleate should be discussed cautiously because current findings are not consistent enough to support a fixed direction of change. Overall, these fatty-acid findings are derived primarily from observational cross-sectional studies and should be interpreted as associative.

Evidence from complex lipids further links lipid dysregulation with the clinical overlap between depression and cognitive impairment. In an untargeted plasma LC–MS study of patients with LLD, compared with the remission phase, levels of tridecanoylcarnitine [Car(13:0)], plasmenyl-phosphatidylcholine [PC(P-16:0/22:6)], and sphingomyelin [SM(d18:1/22:0)] were all markedly downregulated during depressive episodes. Notably, Car(13:0) and PC(P-16:0/22:6) were linked with both depressive severity and cognitive performance, and may partially explain the association between depressive symptoms and cognitive impairment (33). These findings are important because acylcarnitines reflect mitochondrial fatty acid handling, plasmalogens are involved in antioxidant defense and membrane integrity, and sphingolipids are closely related to myelin and white matter biology. Collectively, these alterations suggest that lipidomic changes in LLD may reflect coordinated disturbances in mitochondrial energy metabolism, membrane homeostasis, and neural systems underlying cognitive function.

Population-based studies of older adults suggest that lipid-related metabolic changes may also be associated with depressive symptom trajectories in later life. In a 12-year aging cohort combined with serum-treated human hippocampal progenitor cell assays, the occurrence of depressive symptoms in older adults was associated with reduced proliferative cell death and increased neuronal differentiation. However, serum from participants with recurrent depressive symptoms induced increased neuronal differentiation accompanied by impaired neuronal morphology (34). Further analysis showed that abnormal neuronal differentiation may be regulated by elevated serum butyrylcarnitine levels and reduced glycerophospholipid/phosphatidylcholine PC35:1(16:0/19:1) levels, suggesting that specific choline phospholipid and acylcarnitine metabolites may participate in the long-term trajectory of depressive symptoms in later life by affecting hippocampal neurogenesis and neuroplasticity (34). As depressive status in this cohort was defined by CES-D symptom thresholds rather than a formal LLD diagnosis, the extent to which these findings generalize to clinically diagnosed LLD remains to be established.

Finally, lipid transport-related proteins provide indirect but complementary evidence of systemic lipid metabolic disruption in LLD. Diniz et al. reported altered plasma levels of apolipoproteins and liver-type fatty acid-binding protein in remitted LLD, including decreased ApoA-IV, ApoA-II, ApoA-I, and FABP-liver and increased ApoB and ApoE (35). Although not lipidomic in design, these findings further support the involvement of disturbed lipid transport and lipoprotein metabolism in the peripheral metabolic alterations associated with LLD.

#### 3.2.2 Supplementary insights from MDD Lipidomics evidence for LLD mechanism research

Since direct lipidomic studies on LLD remain relatively limited, findings from peripheral lipidomics in MDD populations can provide indirect insights into lipid metabolism abnormalities in LLD. However, these results cannot be simply extrapolated to LLD. Existing MDD studies show that alterations in peripheral lipid profiles may involve multiple classes of lipid molecules, including glycerophospholipids, sphingolipids, fatty acids, and acylcarnitines. Among these, elevated oxidized fatty acids, reduced acylcarnitines, and changes in membrane lipid molecules such as LPC, LPE, and PE have been relatively commonly reported (36–39).

Some studies also suggest that fatty acid composition may be related to oxidative stress, HPA axis function, and antidepressant treatment response. For example, in drug-naïve MDD patients, red blood cell membrane fatty acid chain length, unsaturation, and peroxidation were elevated, and the negative correlation of fatty acid unsaturation and peroxidation with cortisol levels was associated with non-response to paroxetine treatment. Low DHA levels may also be related to poor treatment response (40). In addition, abnormalities in sphingolipids and ceramides are considered to be involved in the pathological processes of MDD (41, 42). MDD patients may also show elevated levels of metabolites such as ceramides and hexosylceramides, and long-chain ceramide levels are positively correlated with age (43). These findings collectively suggest that abnormal lipid metabolism may be linked to inflammation, oxidative stress, and neuroendocrine abnormalities in depression.

Overall, MDD lipidomic studies offer mechanistic clues for LLD, but their findings should be interpreted cautiously given the aging-related and clinical complexity of LLD. Future LLD-specific longitudinal lipidomic and multi-omics studies are needed to clarify the role of lipid alterations in disease progression and cognitive impairment.

## 4 Potential central lipid alterations in LLD

### 4.1 Phospholipid metabolism in LLD

In recent years, abnormal central lipid metabolism in the pathological of LLD has gradually attracted attention. The central nervous system represents one of the most lipid-abundant tissues in humans, with lipids constitute over half of the brain’s dry mass. Cholesterol, fatty acids, phospholipids, and sphingolipids are not only important structural components of neuronal, glial, and myelin membranes, but also participate in various processes, including cell membrane stability, synapse formation, neurotransmission, lipid raft signal transduction, blood–brain barrier integrity, neuroinflammatory responses, and injury repair (44). Therefore, disturbances in central lipid metabolism may contribute to the development of LLD by affecting neuronal membrane homeostasis, glial cell function, myelin integrity, and synaptic plasticity.

Abnormal phospholipid metabolism is a central lipid alteration that has received considerable attention in LLD. Phospholipids are core components of neuronal and myelin membranes, and their content and fatty acid chain composition can affect membrane fluidity, receptor distribution, synaptic signal transmission, and myelin integrity (45). In the brain, phospholipid composition depends on *de novo* synthesis and fatty acid chain remodeling to maintain dynamic balance; impairment of this homeostasis may lead to membrane structural instability, synaptic dysfunction, and enhanced inflammatory signaling, and is associated with neurodegenerative changes and cognitive impairment (46–48).

Direct human evidence for abnormal brain phospholipid metabolism in LLD remains extremely limited. Harper et al. used 4 Tesla phosphorus-31 magnetic resonance spectroscopy three-dimensional chemical shift imaging to compare 10 unmedicated LLD patients and 11 healthy elderly controls. They found elevated glycerophosphoethanolamine levels in the white matter of LLD patients, whereas glycerophosphocholine levels showed no significant differences (49). Glycerophosphoethanolamine is an important product of membrane phospholipid catabolism, and its elevation suggests accelerated membrane phospholipid turnover or enhanced catabolism in the white matter of LLD patients. Given the role of white matter integrity in emotion regulation, executive function, and cognition, this finding suggests that altered membrane lipid metabolism and increased phospholipid breakdown may link LLD to aging-related neurodegenerative changes. Animal study also suggests region-specific changes in central glycerophospholipid metabolism in aging-related depression. In a natural aging LOD rat model investigated in the context of luteolin treatment, phosphatidylserine, phosphatidylcholine, and phosphatidylethanolamine were decreased in the hippocampus but increased in the prefrontal cortex (50). Given the pharmacological intervention design, these region-specific alterations may reflect treatment effects rather than spontaneous disease-related changes. These regionally divergent changes, together with their behavioral correlations, suggest that glycerophospholipid dysregulation may be related to emotional and cognitive abnormalities in aging-related depression.

### 4.2 APOE-related pathways in LLD

APOE-related pathways may provide a potential genetic link between lipid metabolism and LLD. In the brain, ApoE is produced mainly by astrocytes and microglia and it regulates cholesterol transport and contributes to neuronal repair, synaptic remodeling, and myelin maintenance. Several observational studies have reported associations between APOE ε4 and depressive symptoms or major depression in older adults (51, 52). However, these epidemiological associations do not establish a direct role for altered central lipid metabolism in LLD. Rather, the relationship between APOE ε4 and LLD may involve a broader range of APOE-related processes, including altered lipid handling, neuroinflammation, amyloid-β metabolism, and blood–brain barrier dysfunction.

Evidence supporting these potential mechanisms comes largely from studies of Alzheimer disease, aging, and experimental models rather than from patients with LLD. ApoE4 can reduce the clearance of amyloid-β (Aβ) across the blood–brain barrier, potentially increasing its retention in the brain (53). Aβ accumulation may, in turn, affect lipid handling in microglia. Experimental studies have shown that Aβ promotes lipid droplet formation and inflammatory responses in microglia, with stronger effects in an APOE4 background (54). APOE4 has also been associated with blood–brain barrier breakdown that appears partly independent of Aβ and tau pathology (55). In addition, animal studies suggest that APOE ε4 may influence neural signaling through impaired GABAergic signaling and enhanced GluN2B phosphorylation (56). Although these findings provide biologically plausible mechanisms through which APOE ε4 could influence vulnerability to LLD, their relevance to LLD remains uncertain, and any proposed link should be considered hypothetical.

Dietary factors may also modify APOE-related effects. In ApoE4 mice, a high-fat diet aggravated lipid accumulation and metabolic abnormalities in neurons and microglia (57). Human evidence remains limited, and available data suggest that dietary effects may differ across APOE genotypes (58).

Overall, current evidence supports a possible association between APOE-related biological pathways and LLD but does not establish a specific central lipid mechanism. APOE ε4 may influence processes involving lipid handling, inflammation, vascular integrity, amyloid metabolism, and neural signaling, several of which are also implicated in aging and neurodegeneration. These pathways may be particularly relevant in older adults, in whom depressive symptoms can coexist with cognitive decline and age-related neurodegenerative changes. Nevertheless, the available evidence is largely indirect. Longitudinal studies integrating brain imaging, central lipid measurements, genetic information, and dietary assessment are needed to clarify whether and how APOE-related lipid pathways contribute to LLD.

## 5 Potential role of dietary lipid regulation in LLD

Dietary lipids are not only sources of energy and structural components of cell membranes, but also important metabolic regulators that influence neuroinflammation, oxidative stress, synaptic plasticity, and neurogenesis. Current research suggests that different types and sources of dietary lipids may have different effects on the development of LLD.

### 5.1 Omega-3 fatty acids and LLD

Polyunsaturated fatty acids (PUFAs) comprise two major classes with distinct biological roles: omega-6 polyunsaturated fatty acids (n-6 PUFAs) and omega-3 polyunsaturated fatty acids (n-3 PUFAs). n-3 PUFAs, including alpha-linolenic acid (ALA) and the long-chain fatty acids eicosapentaenoic acid (EPA) and docosahexaenoic acid (DHA), have received particular attention in relation to depressive outcomes. ALA is obtained mainly from plant foods, whereas EPA and DHA are derived largely from marine sources or from limited endogenous conversion of ALA (59). Observational studies have linked lower omega-3 status with mood disorders, but such associations cannot establish whether low omega-3 exposure precedes depression or instead reflects dietary changes accompanying depressive symptoms (60).

Currently, findings do not support a general protective effect. In a large long-term randomized controlled trial among adults aged 50 years and older, daily supplementation with 1 g of marine-derived omega-3 fatty acids did not reduce the risk of depression and instead was associated with a modest but statistically significant increase in risk (61). Consistent with this finding, the systematic review by Deane et al. concluded that long-chain omega-3 probably has little or no effect on depression or anxiety symptoms, while evidence for other PUFA exposures was limited or of low certainty (62).

Evidence in patients with diagnosed depression is more heterogeneous. Kelaiditis et al. reported that formulations in which EPA accounted for at least 60% of total EPA + DHA, at total doses of 1–2 g/day, were associated with greater reductions in depression severity, although effects varied according to dose and study characteristics (63). These findings suggest that the antidepressant effects of omega-3 fatty acids may depend not only on EPA proportion and dose, but also on individual biological characteristics. Poor baseline omega-3 status may leave greater scope for benefit from supplementation, whereas genetic variation in PUFA metabolism, such as polymorphisms in the FADS gene cluster that influence tissue long-chain PUFA levels, may contribute to interindividual differences in treatment response (63). A small 8-week randomized controlled trial in 46 older women with depression also reported reduced GDS scores and improved SF-36 scores after supplementation with EPA-rich n-3 PUFA (64). However, the small sample size, restricted female nursing-home population, short duration, and heterogeneity across intervention studies limit the generalizability of these findings. They therefore suggest a possible adjunctive effect in selected patients rather than evidence for routine use across older adults.

Mechanistically, EPA and DHA can be incorporated into membrane phospholipids and serve as precursors of lipid mediators involved in inflammatory resolution (65). Animal studies provide some support for this hypothesis. Previous studies have demonstrated that omega-3 fatty acid supplementation can alleviate chronic stress-induced depression-like behaviors and improve oxidative damage and lipid metabolism disturbances in brain tissue (66, 67). Conversely, omega-3 fatty acid deficiency can lead to decreased DHA levels in the brain and increased depression-like behaviors (68). These results suggest that altered brain lipid composition caused by n-3 PUFA deficiency may contribute to depression-related pathophysiological processes. In addition, omega-3 PUFA-enriched diets may exert antidepressant-like effects by promoting hippocampal neurogenesis and upregulating brain-derived neurotrophic factor (BDNF) and synaptophysin expression (69). These findings provide biological plausibility but do not demonstrate clinical efficacy or establish that the same pathways mediate LLD in humans.

Overall, the strongest clinical evidence does not support omega-3 supplementation as a universal strategy for the prevention of depression in later life. Whether EPA-enriched formulations benefit particular older patients with diagnosed depression remains uncertain and requires larger LLD-specific trials with adequate duration and prespecified subgroup analyses.

### 5.2 High-fat diet and LLD

A high-fat diet is a broad and heterogeneous exposure that varies in fatty-acid composition, food source, processing method, and total energy intake. Human evidence specifically linking high-fat intake to LLD is limited and largely observational. Payne et al. reported that patients with LLD tended to consume more saturated fat and cholesterol and also had a higher body mass index (70). In a separate study of 54 patients aged 60 years and older with vascular depression, high-fat dairy intake assessed by a food-frequency questionnaire was correlated with greater white matter lesion volume (71). These studies identify associations but do not establish the temporal direction of the relationship or an independent effect of dietary fat.

Several explanations should therefore be considered. Depressive symptoms may alter appetite, food choice, and reporting of intake, creating the possibility of reverse causality. Socioeconomic status, physical activity, smoking, and other lifestyle factors may be related to both diet and depression, while obesity, dyslipidemia, cardiovascular disease, diabetes, and other comorbidities may confound or mediate the observed relationships. In addition, food-frequency questionnaires are vulnerable to recall error and dietary misclassification. The small samples and selected clinical populations further limit generalizability. Consequently, current clinical data do not justify describing high-fat intake as an established causal risk factor or therapeutic target for LLD.

Animal experiments provide a different type of evidence. Long-term high-fat feeding has produced depression-like behaviors together with alterations in lipid metabolism, inflammatory activation, and microglial function in experimental models (72–74). Such findings can generate hypotheses about pathways linking metabolic disturbance with mood-related behavior.

Thus, the current literature supports an association-based interpretation of high-fat dietary exposure. Future research should include longitudinal cohorts with repeated dietary assessments and better control for cardiometabolic, socioeconomic, and lifestyle confounders. Well-designed intervention studies are also needed before causal conclusions can be drawn.

### 5.3 Dietary lipid quality and LLD

Beyond total fat intake, lipid quality, food sources, and processing methods may be more relevant to LLD. Because older adults often have chronic inflammation, oxidative stress, vascular dysfunction, and metabolic impairment, they may be particularly sensitive to different lipid exposures.

Evidence from the Greek EPIC cohort indicates that fatty acid type may be more relevant to depressive symptoms than total fat intake. Among 610 adults aged ≥60 years followed for 6–13 years, higher Geriatric Depression Scale scores were associated with lower monounsaturated fatty acid and olive oil intake, but with higher seed oil intake. By contrast, fish and seafood consumption showed no significant association with depression scores, nor were total energy, total fat, or saturated fat intakes associated with depression scores (75). As seed oils are major dietary sources of linoleic acid-rich n-6 PUFAs, whereas fish and seafood provide long-chain n-3 PUFAs, these findings suggest that associations with depressive symptoms may vary according to PUFA subtype and food source rather than total PUFA intake alone. Yet because n-6 PUFA intake was not evaluated separately, the contribution of specific PUFA subclasses remains unclear.

Lipid processing methods may also affect mental health. Wang et al. found in a large-scale cohort study that frequent consumption of fried foods, particularly fried potatoes, was linked with increased risks of anxiety and depression. Animal experiments further showed that acrylamide, a contaminant related to fried foods, disrupted cerebral sphingolipid and phospholipid metabolism, enhanced lipid peroxidation and oxidative stress, upregulated pro-inflammatory lipid mediators, and induced anxiety- and depression-like behaviors in zebrafish (76).

Lipid exposure should be considered within the overall dietary pattern. The Western diet, characterized by high saturated fat, refined carbohydrates and energy-dense processed foods, may impair cognitive and emotional function through metabolic dysfunction, inflammation, gut-brain axis disruption, and HPA axis dysregulation (77, 78). As these effects reflect multiple interacting dietary factors, existing data do not support attributing LLD risk solely to fat intake.

Taken together, current evidence suggests that the composition, source, and broader dietary context of lipid intake may be more informative than total fat intake alone, but the available findings are predominantly associative. They are insufficient to support specific lipid-based recommendations for the prevention or treatment of LLD.

### 5.4 Limitations and future research directions

Several limitations should be considered. LLD-specific lipidomic evidence remains limited, and many findings are derived from MDD or mixed-age populations. Most available studies have small samples and use cross-sectional or case–control designs, which limits causal inference. Differences in lipid measures, biological samples, analytical methods, and depression definitions also reduce comparability across studies. In addition, the limited racial and ethnic diversity of current cohorts may restrict generalizability. Medication use, diet, and common age-related comorbidities, including obesity, diabetes, and cardiovascular disease, may further confound lipid-LLD associations.

Future research should focus on large, multicenter longitudinal studies with diverse populations. These studies should include well-defined clinical features, such as age at onset, disease stage, and treatment status. This may help clarify the temporal relationship between lipid abnormalities and LLD and provide stronger evidence for possible causal links. Multi-omics approaches may also be useful. Combining lipidomics with genomics, proteomics, and neuroimaging could help examine the relationship between peripheral lipid changes and central biological processes. It may also help identify pathways for further mechanistic study. Intervention studies on lipid-related pathways are still limited. In particular, the effects of n-3 PUFAs and dietary lipid quality should be evaluated in well-designed trials involving older adults with depression. These studies may help determine whether lipid-related biomarkers and interventions are useful for risk assessment, disease monitoring, and personalized management of LLD.

## 6 Conclusion

Current evidence suggests that lipid abnormalities are associated with LLD, but their causal role remains uncertain. Findings vary across lipid measures, study populations, and clinical characteristics, highlighting the heterogeneity of LLD and the need for stratified analyses. Evidence for central lipid alterations remains limited, and current dietary studies, including those of omega-3 supplementation, do not support specific lipid-based interventions for the prevention or treatment of LLD. Larger longitudinal and LLD-specific studies are needed to clarify these associations and their clinical relevance.

## References

[1] Alexopoulos GS. Depression in the elderly. Lancet. (2005) 365:1961–70. doi: 10.1016/S0140-6736(05)66665-2, 15936426

[2] Institute for Health Metrics and Evaluation. 2021 Global Burden of Disease (GBD) [Online Database]. Seattle: Institute for Health Metrics and Evaluation; (2024). Available online at: https://vizhub.healthdata.org/gbd-results/ (Accessed August 13, 2025).

[3] World Health Organization. Depressive disorder (depression). (2025). Available online at: https://www.who.int/news-room/fact-sheets/detail/depression. (Accessed July 2, 2026).

[4] Abdoli N Salari N Darvishi N Jafarpour S Solaymani M Mohammadi M . The global prevalence of major depressive disorder (MDD) among the elderly: a systematic review and meta-analysis. Neurosci Biobehav Rev. (2022) 132:1067–73. doi: 10.1016/j.neubiorev.2021.10.041, 34742925

[5] Yu X. Expert consensus on diagnosis and treatment of late life depression (2025 edition). Chin J Psychiatry. (2025) 58:592–602. doi: 10.3760/cma.j.cn113661-20250213-00066

[6] Lal S Gunji S Ahluwalia P Kolhe R Bollag WB Hill WD . Lipid metabolism in age-related musculoskeletal disorders: insights into sarcopenia and osteoporosis. BMC Biol. (2025) 23:265. doi: 10.1186/s12915-025-02383-9, 40846955 PMC12374409

[7] Senko D Zavolskova M Efimova O Morozova A Zorkina Y Kostyuk G . Comparative lipidomic alterations in the anterior cingulate cortex in schizophrenia and major depression. Schizophr Res. (2026) 287:147–55. doi: 10.1016/j.schres.2025.12.005, 41412002

[8] Wei X Qin W. m6A-metabolite axes in depression: METTL14 network dysregulation and RBM15 lipid protection. J Affect Disord. (2026) 398:120981. doi: 10.1016/j.jad.2025.120981, 41429321

[9] Jellinger KA. The enigma of vascular depression in old age: a critical update. J Neural Transm (Vienna). (2022) 129:961–76. doi: 10.1007/s00702-022-02521-5, 35705878

[10] Chae WR Fuentes-Casañ M Gutknecht F Ljubez A Gold SM Wingenfeld K . Early-onset late-life depression: association with body mass index, obesity, and treatment response. Compr Psychoneuroendocrinol. (2021) 8:100096. doi: 10.1016/j.cpnec.2021.100096, 35757669 PMC9216262

[11] Toth MJ Tchernof A. Lipid metabolism in the elderly. Eur J Clin Nutr. (2000) 54:S121–5. doi: 10.1038/sj.ejcn.1601033, 11041083

[12] Mesa-Herrera F Taoro-González L Valdés-Baizabal C Diaz M Marín R. Lipid and lipid raft alteration in aging and neurodegenerative diseases: a window for the development of new biomarkers. Int J Mol Sci. (2019) 20:3810. doi: 10.3390/ijms20153810, 31382686 PMC6696273

[13] Söderberg M Edlund C Kristensson K Dallner G. Lipid compositions of different regions of the human brain during aging. J Neurochem. (1990) 54:415–23. doi: 10.1111/j.1471-4159.1990.tb01889.x, 2299344

[14] Mota-Martorell N Andrés-Benito P Martín-Gari M Galo-Licona JD Sol J Fernández-Bernal A . Selective brain regional changes in lipid profile with human aging. Geroscience. (2022) 44:763–83. doi: 10.1007/s11357-022-00527-1, 35149960 PMC9135931

[15] Schaakxs R Comijs HC Lamers F Kok RM Beekman ATF Penninx BWJH. Associations between age and the course of major depressive disorder: a 2-year longitudinal cohort study. Lancet Psychiatry. (2018) 5:581–90. doi: 10.1016/S2215-0366(18)30166-4, 29887519

[16] Faizan AYW Faizan S Ilyas S Mudassar H Khalid H Naseer S . Prevalence of metabolic syndrome and its components in patients with major depressive disorder. Cureus. (2025) 17:e83066. doi: 10.7759/cureus.83066, 40432661 PMC12107968

[17] Virtanen M Ferrie JE Akbaraly T Tabak A Jokela M Ebmeier KP . Metabolic syndrome and symptom resolution in depression: a 5-year follow-up of older adults. J Clin Psychiatry. (2017) 78:e1–7. doi: 10.4088/JCP.15m10399, 28129497

[18] Marijnissen RM Vogelzangs N Mulder ME van den Brink RHS Comijs HC Oude Voshaar RC. Metabolic dysregulation and late-life depression: a prospective study. Psychol Med. (2017) 47:1041–52. doi: 10.1017/S0033291716003196, 27938429

[19] Vogelzangs N Beekman AT Boelhouwer IG Beekman ATF Bandinelli S Milaneschi Y . Metabolic depression: a chronic depressive subtype? Findings from the InCHIANTI study of older persons. J Clin Psychiatry. (2011) 72:598–604. doi: 10.4088/JCP.10m06559, 21535996 PMC6232848

[20] Dekker IP Marijnissen RM Giltay EJ van der Mast RC Oude Voshaar RC Rhebergen D . The role of metabolic syndrome in late-life depression over 6 years: the NESDO study. J Affect Disord. (2019) 257:735–40. doi: 10.1016/j.jad.2019.07.060, 31386966

[21] Kim JM Stewart R Kim SW Yang SJ Shin IS Yoon JS. Vascular risk factors and incident late-life depression in a Korean population. Br J Psychiatry. (2006) 189:26–30. doi: 10.1192/bjp.bp.105.015032, 16816302

[22] Kim JM Stewart R Kim SW Shin IS Yang SJ Yoon JS. Cholesterol and serotonin transporter polymorphism interactions in late-life depression. Neurobiol Aging. (2011) 32:336–43. doi: 10.1016/j.neurobiolaging.2009.02.017, 19329228

[23] Ancelin ML Carrière I Boulenger JP Malafosse A Stewart R Cristol JP . Gender and genotype modulation of the association between lipid levels and depressive symptomatology in community-dwelling elderly (the ESPRIT study). Biol Psychiatry. (2010) 68:125–32. doi: 10.1016/j.biopsych.2010.04.011, 20537614

[24] Liang Y Yan Z Cai C Jiang H Song A Qiu C. Association between lipid profile and depressive symptoms among Chinese older people: mediation by cardiovascular diseases? Int J Behav Med. (2014) 21:590–6. doi: 10.1007/s12529-013-9358-2, 24136399

[25] Gu C Long Z Fang Z Wu J Yang F Luo W. Nonlinear association between the ratio of non-high-density lipoprotein cholesterol to high-density lipoprotein cholesterol (NHHR), residual cholesterol (RC), and late-life depression: the potential mediating role of cardiovascular metabolic diseases in the elderly population of China. BMC Geriatr. (2026) 26:245. doi: 10.1186/s12877-026-07029-1, 41606490 PMC12924423

[26] Huang X Yuan Sun MM Zhang XY. Prevalence and clinical correlates of abnormal lipid metabolism in older Chinese patients with first-episode drug-naïve major depressive disorder. BMC Psychiatry. (2024) 24:534. doi: 10.1186/s12888-024-05967-x, 39054520 PMC11270971

[27] Li B Li M Li M Wang X Kang M Zhang G . Prevalence and associated factors of psychotic symptoms in young first-episode, drug-naïve major depressive disorder patients with abnormal lipid metabolism. Eur J Med Res. (2025) 31:131. doi: 10.1186/s40001-025-03718-6, 41423626 PMC12837187

[28] Chourpiliadis C Zeng Y Lovik A Wei D Valdimarsdóttir U Song H . Metabolic profile and long-term risk of depression, anxiety, and stress-related disorders. JAMA Netw Open. (2024) 7:e244525. doi: 10.1001/jamanetworkopen.2024.4525, 38564219 PMC10988352

[29] Shin HY Kang G Kang HJ Kim SW Shin IS Yoon JS . Relationships between high-density lipoprotein cholesterol and depressive symptoms: findings of the Korean National Health and nutrition examination survey (KNHANES). Psychiatry Res. (2016) 241:172–4. doi: 10.1016/j.psychres.2016.05.003, 27179182

[30] Milaneschi Y Cesari M Simonsick EM Vogelzangs N Kanaya AM Yaffe K . Lipid peroxidation and depressed mood in community-dwelling older men and women. PLoS One. (2013) 8:e65406. doi: 10.1371/journal.pone.0065406, 23776478 PMC3679197

[31] Diniz BS Mendes-Silva AP Silva LB Bertola L Vieira MC Ferreira JD . Oxidative stress markers imbalance in late-life depression. J Psychiatr Res. (2018) 102:29–33. doi: 10.1016/j.jpsychires.2018.02.023, 29574402

[32] Gao Y Hu JZ Wen ZP Dong T du XZ Liu ZF . Metabolomic insights into late-life depression: a systematic review. BMC Geriatr. (2025) 25:618. doi: 10.1186/s12877-025-06256-2, 40804658 PMC12344915

[33] Wang Z Chen B Xu D Zhu K Chen J Tian S . Plasma metabolomics identifies lipid mediators linking depression and cognitive decline in late-life depression. J Affect Disord. (2026) 400:121094. doi: 10.1016/j.jad.2025.121094, 41483882

[34] Du Preez A Lefèvre-Arbogast S González-Domínguez R Houghton V de Lucia C Low DY . Impaired hippocampal neurogenesis in vitro is modulated by dietary-related endogenous factors and associated with depression in a longitudinal ageing cohort study. Mol Psychiatry. (2022) 27:3425–40. doi: 10.1038/s41380-022-01644-1, 35794184 PMC7613865

[35] Diniz BS Lin CW Sibille E Tseng G Lotrich F Aizenstein HJ . Circulating biosignatures of late-life depression (LLD): towards a comprehensive, data-driven approach to understanding LLD pathophysiology. J Psychiatr Res. (2016) 82:1–7. doi: 10.1016/j.jpsychires.2016.07.006, 27447786 PMC9344393

[36] He L Duan N Wang C Shan R Li J Wang L . Lipidomic analyses reveal the dysregulation of oxidized fatty acids (OxFAs) and acyl-carnitines (CARs) in major depressive disorder: a case-control study. BMC Psychiatry. (2025) 25:752. doi: 10.1186/s12888-025-07191-7, 40751139 PMC12317606

[37] Liu X Li J Zheng P Zhao X Zhou C Hu C . Plasma lipidomics reveals potential lipid markers of major depressive disorder. Anal Bioanal Chem. (2016) 408:6497–507. doi: 10.1007/s00216-016-9768-5, 27457104

[38] Liu X Zheng P Zhao X Zhang Y Hu C Li J . Discovery and validation of plasma biomarkers for major depressive disorder classification based on liquid chromatography-mass spectrometry. J Proteome Res. (2015) 14:2322–30. doi: 10.1021/acs.jproteome.5b00144, 25784130

[39] Jansen R Milaneschi Y Schranner D Kastenmuller G Arnold M Han X . The metabolome-wide signature of major depressive disorder. Mol Psychiatry. (2024) 29:3722–33. doi: 10.1038/s41380-024-02613-6, 38849517

[40] Mocking RJ Verburg HF Westerink AM Mocking RJT Assies J Vaz FM . Fatty acid metabolism and its longitudinal relationship with the hypothalamic-pituitary-adrenal axis in major depression: associations with prospective antidepressant response. Psychoneuroendocrinology. (2015) 59:1–13. doi: 10.1016/j.psyneuen.2015.04.027, 26010860

[41] Wang J Hu X Li Y Li S Wang T Wang D . Impaired lipid homeostasis and elevated lipid oxidation of erythrocyte membrane in adolescent depression. Redox Biol. (2025) 80:103491. doi: 10.1016/j.redox.2025.103491, 39809016 PMC11780951

[42] Bisle E Haange SB Rojas R Behnke A Karabatsiakis A Gumpp A . Serum metabolomics in women with major depressive disorder: associations with mitochondrial function, inflammation, and oxidative stress. Psychiatry Res. (2025) 351:116569. doi: 10.1016/j.psychres.2025.116569, 40480066

[43] Brunkhorst-Kanaan N Klatt-Schreiner K Hackel J Schröter K Trautmann S Hahnefeld L . Targeted lipidomics reveal derangement of ceramides in major depression and bipolar disorder. Metabolism. (2019) 95:65–76. doi: 10.1016/j.metabol.2019.04.002, 30954559

[44] Vanherle S Loix M Miron VE Hendriks JJA Bogie JFJ. Lipid metabolism, remodelling and intercellular transfer in the CNS. Nat Rev Neurosci. (2025) 26:214–31. doi: 10.1038/s41583-025-00908-3, 39972160

[45] Tavasoli M Lahire S Reid T Brodovsky M McMaster CR. Genetic diseases of the Kennedy pathways for membrane synthesis. J Biol Chem. (2020) 295:17877–86. doi: 10.1074/jbc.REV120.013529, 33454021 PMC7762932

[46] Sagy-Bross C Kasianov K Solomonov Y Braiman A Friedman A Hadad N . The role of cytosolic phospholipase A2 α in amyloid precursor protein induction by amyloid beta1-42: implication for neurodegeneration. J Neurochem. (2015) 132:559–71. doi: 10.1111/jnc.13012, 25533654

[47] Sanchez-Mejia RO Newman JW Toh S Yu GQ Zhou Y Halabisky B . Phospholipase A2 reduction ameliorates cognitive deficits in a mouse model of Alzheimer's disease. Nat Neurosci. (2008) 11:1311–8. doi: 10.1038/nn.2213, 18931664 PMC2597064

[48] Ham HJ Han SB Yun J Yeo IJ Ham YW Kim SH . Bee venom phospholipase A2 ameliorates amyloidogenesis and neuroinflammation through inhibition of signal transducer and activator of transcription-3 pathway in Tg2576 mice. Transl Neurodegener. (2019) 8:26. doi: 10.1186/s40035-019-0167-7, 31592103 PMC6774221

[49] Harper DG Jensen JE Ravichandran C Sivrioglu Y Silveri M Iosifescu DV . Tissue-specific differences in brain phosphodiesters in late-life major depression. Am J Geriatr Psychiatry. (2014) 22:499–509. doi: 10.1016/j.jagp.2012.08.005, 23567437 PMC3749264

[50] Wu X Xu H Zeng N Li H Yao G Liu K . Luteolin alleviates depression-like behavior by modulating glycerophospholipid metabolism in the hippocampus and prefrontal cortex of LOD rats. CNS Neurosci Ther. (2024) 30:e14455. doi: 10.1111/cns.14455, 37715585 PMC10916417

[51] Skoog I Waern M Duberstein P Blennow K Zetterberg H Börjesson-Hanson A . A 9-year prospective population-based study on the association between the APOE*E4 allele and late-life depression in Sweden. Biol Psychiatry. (2015) 78:730–6. doi: 10.1016/j.biopsych.2015.01.006, 25708227

[52] Yen YC Rebok GW Gallo JJ Yang MJ Lung FW Shih CH. ApoE4 allele is associated with late-life depression: a population-based study. Am J Geriatr Psychiatry. (2007) 15:858–68. doi: 10.1097/JGP.0b013e3180f63373, 17911363

[53] Deane R Sagare A Hamm K Parisi M Lane S Finn MB . apoE isoform-specific disruption of amyloid beta peptide clearance from mouse brain. J Clin Invest. (2008) 118:4002–13. doi: 10.1172/JCI36663, 19033669 PMC2582453

[54] Haney MS Pálovics R Munson CN Long C Johansson PK Yip O . APOE4/4 is linked to damaging lipid droplets in Alzheimer's disease microglia. Nature. (2024) 628:154–61. doi: 10.1038/s41586-024-07185-7, 38480892 PMC10990924

[55] Montagne A Nation DA Sagare AP Barisano G Sweeney MD Chakhoyan A . APOE4 leads to blood-brain barrier dysfunction predicting cognitive decline. Nature. (2020) 581:71–6. doi: 10.1038/s41586-020-2247-3, 32376954 PMC7250000

[56] Zhang J Lin L Dai X Xiao N' Ye Q Chen X. ApoE4 increases susceptibility to stress-induced age-dependent depression-like behavior and cognitive impairment. J Psychiatr Res. (2021) 143:292–301. doi: 10.1016/j.jpsychires.2021.09.029, 34530340

[57] Wang C Lu J Sha X Qiu Y Chen H Yu Z. TRPV1 regulates ApoE4-disrupted intracellular lipid homeostasis and decreases synaptic phagocytosis by microglia. Exp Mol Med. (2023) 55:347–63. doi: 10.1038/s12276-023-00935-z, 36720919 PMC9981624

[58] Al Shamsi HSS Gardener SL Rainey-Smith SR Pedrini S Sohrabi HR Taddei K . The moderating effect of diet on the relationship between depressive symptoms and Alzheimer's disease-related blood-based biomarkers. Neurobiol Aging. (2025) 147:213–22. doi: 10.1016/j.neurobiolaging.2025.01.003, 39837054

[59] Grosso G Galvano F Marventano S Malaguarnera M Bucolo C Drago F . Omega-3 fatty acids and depression: scientific evidence and biological mechanisms. Oxidative Med Cell Longev. (2014) 2014:313570. doi: 10.1155/2014/313570, 24757497 PMC3976923

[60] Komori T. The effects of phosphatidylserine and omega-3 fatty acid-containing supplement on late life depression. Ment Illn. (2015) 7:5647. doi: 10.4081/mi.2015.5647, 26266022 PMC4508628

[61] Okereke OI Vyas CM Mischoulon D Chang G Cook NR Weinberg A . Effect of long-term supplementation with marine omega-3 fatty acids vs placebo on risk of depression or clinically relevant depressive symptoms and on change in mood scores: a randomized clinical trial. JAMA. (2021) 326:2385–94. doi: 10.1001/jama.2021.21187, 34932079 PMC8693224

[62] Deane KHO Jimoh OF Biswas P O'Brien A Hanson S Abdelhamid AS . Omega-3 and polyunsaturated fat for prevention of depression and anxiety symptoms: systematic review and meta-analysis of randomised trials. Br J Psychiatry. (2021) 218:135–42. doi: 10.1192/bjp.2019.234, 31647041

[63] Kelaiditis CF Gibson EL Dyall SC. Effects of long-chain omega-3 polyunsaturated fatty acids on reducing anxiety and/or depression in adults: a systematic review and meta-analysis of randomised controlled trials. Prostaglandins Leukot Essent Fat Acids. (2023) 192:102572. doi: 10.1016/j.plefa.2023.102572, 37028202

[64] Rondanelli M Giacosa A Opizzi A Pelucchi C la Vecchia C Montorfano G . Long chain omega 3 polyunsaturated fatty acids supplementation in the treatment of elderly depression: effects on depressive symptoms, on phospholipids fatty acids profile and on health-related quality of life. J Nutr Health Aging. (2011) 15:37–44. doi: 10.1007/s12603-011-0011-y, 21267525 PMC12879681

[65] Farioli Vecchioli S Sacchetti S Nicolis di Robilant V Cutuli D. The role of physical exercise and omega-3 fatty acids in depressive illness in the elderly. Curr Neuropharmacol. (2018) 16:308–26. doi: 10.2174/1570159X15666170912113852 PMC5843982 28901279

[66] Réus GZ Maciel AL Abelaira HM de Moura AB de Souza TG Dos Santos TR . Omega-3 and folic acid act against depressive-like behavior and oxidative damage in the brain of rats subjected to early- or late-life stress. Nutrition. (2018) 53:120–33. doi: 10.1016/j.nut.2018.03.006 29783176

[67] Tang M Jiang P Li H Liu Y Cai H Dang R . Fish oil supplementation alleviates depressant-like behaviors and modulates lipid profiles in rats exposed to chronic unpredictable mild stress. BMC Complement Altern Med. (2015) 15:239. doi: 10.1186/s12906-015-0778-1, 26183327 PMC4504181

[68] DeMar JC Jr Ma K Bell JM Igarashi M Greenstein D Rapoport SI. One generation of n-3 polyunsaturated fatty acid deprivation increases depression and aggression test scores in rats. J Lipid Res. (2006) 47:172–80. doi: 10.1194/jlr.M500362-JLR200, 16210728

[69] Venna VR Deplanque D Allet C Belarbi K Hamdane M Bordet R. PUFA induce antidepressant-like effects in parallel to structural and molecular changes in the hippocampus. Psychoneuroendocrinology. (2009) 34:199–211. doi: 10.1016/j.psyneuen.2008.08.025, 18848400

[70] Payne ME Hybels CF Bales CW Steffens DC. Vascular nutritional correlates of late-life depression. Am J Geriatr Psychiatry. (2006) 14:787–95. doi: 10.1097/01.JGP.0000203168.28872.21, 16943175

[71] Payne ME Haines PS Chambless LE Anderson JJB Steffens DC. Food group intake and brain lesions in late-life vascular depression. Int Psychogeriatr. (2007) 19:295–305. doi: 10.1017/S1041610206004431, 17054820

[72] Yu H Qin X Yu Z Chen Y Tang L Shan W. Effects of high-fat diet on the formation of depressive-like behavior in mice. Food Funct. (2021) 12:6416–31. doi: 10.1039/d1fo00044f, 34076000

[73] Li Y Cheng Y Zhou Y du H Zhang C Zhao Z . High fat diet-induced obesity leads to depressive and anxiety-like behaviors in mice via AMPK/mTOR-mediated autophagy. Exp Neurol. (2022) 348:113949. doi: 10.1016/j.expneurol.2021.113949, 34902357

[74] Liu C Li Z Ji JJ Liu K Samsom JN Li T . Single-cell RNA sequencing uncovers molecular features underlying microglial lipid accumulation and depression-related behaviors in high-fat diet mouse model of obesity. Neuropsychopharmacology. (2026) 51:577–87. doi: 10.1038/s41386-025-02273-2, 41198848 PMC12824154

[75] Kyrozis A Psaltopoulou T Stathopoulos P Trichopoulos D Vassilopoulos D Trichopoulou A. Dietary lipids and geriatric depression scale score among elders: the EPIC-Greece cohort. J Psychiatr Res. (2009) 43:763–9. doi: 10.1016/j.jpsychires.2008.09.003, 18952225

[76] Wang A Wan X Zhuang P Jia W Ao Y Liu X . High fried food consumption impacts anxiety and depression due to lipid metabolism disturbance and neuroinflammation. Proc Natl Acad Sci USA. (2023) 120:e2221097120. doi: 10.1073/pnas.2221097120, 37094155 PMC10160962

[77] López-Taboada I González-Pardo H Conejo NM. Western diet: implications for brain function and behavior. Front Psychol. (2020) 11:564413. doi: 10.3389/fpsyg.2020.564413, 33329193 PMC7719696

[78] Matison AP Mather KA Flood VM Reppermund S. Associations between nutrition and the incidence of depression in middle-aged and older adults: a systematic review and meta-analysis of prospective observational population-based studies. Ageing Res Rev. (2021) 70:101403. doi: 10.1016/j.arr.2021.101403, 34246793

